# Electrophysiological Responses of *Trissolcus japonicus*, *T. basalis*, and *T. oenone* (Hymenoptera: Scelionidae) to Volatile Compounds Associated with New Zealand Stink Bugs (Hemiptera: Pentatomidae)

**DOI:** 10.1007/s10886-024-01533-7

**Published:** 2024-08-03

**Authors:** Thomas E. Saunders, Lee-Anne M. Manning, Gonzalo A. Avila, Gregory I. Holwell, Kye Chung Park

**Affiliations:** 1 Te Kura Mātauranga Koiora, School of Biological Sciences, Waipapa Taumata Rau, University of Auckland, Private Bag 92019, Auckland, New Zealand; 2 The New Zealand Institute for Plant & Food Research Limited, Auckland, New Zealand; 3 Better Border Biosecurity, http://www.b3nz.org

**Keywords:** Electrophysiology, Host Range, Non-Target Risks, Host Chemistry, Parasitism

## Abstract

Parasitoid biological control agents rely heavily on olfaction to locate their hosts. Chemical cues associated with hosts and non-hosts are known to influence the expression of host preferences and host-specificity. A better understanding of how and why parasitoids attack some species and not others, based on volatile organic compounds associated with potential hosts, can provide key information on the parasitoid’s host preferences, which could be applied to pre-release risk assessments for classical biological control agents. Electrophysiological techniques such as electroantennography (EAG) and GC-EAD (gas chromatography coupled with electroantennographic detection) are widely used to identify bioactive semiochemicals. But the application of these techniques to understanding how chemical ecological cues mediate parasitoid host specificity has not been as thoroughly explored. We conducted GC-EAD and EAG studies to identify olfactory-active compounds associated with adult females of nine stink bug species from Aotearoa/New Zealand on the antennae of three closely related parasitoid species: *Trissolcus japonicus* Ashmead, a pre-emptively (= proactively) approved biocontrol agent against brown marmorated stink bug; *T. basalis* (Wollaston), a biocontrol agent introduced against *Nezara viridula* L. in 1949; and *T. oenone* Johnson, a native Australasian pentatomid parasitoid. Eight compounds associated with stink bugs elicited antennal responses from all three parasitoids, and we were able to identify seven of these. (*E*)-2-hexenal, (*E*)-4-oxo-2-hexenal, (*E*)-2-octenal and (*E*)-2-decenal generally elicited stronger responses in the three parasitoids, while *n*-tridecane, *n*-dodecane, and (*E*)-2-decenyl acetate elicited weaker responses. We discuss how and why the results from electrophysiological experiments can be applied to non-target risk assessments within biological control programmes.

## Introduction

Parasitic Hymenoptera have evolved to rely on olfaction for finding hosts (Vinson 1998). Parasitoid antennal sensilla often contain olfactory receptor neurons attuned to a relatively narrow range of volatile organic compounds associated with their hosts, such as cuticular hydrocarbons or compounds in waste products (Vet and Dicke 1992; Blomquist and Ginzel 2021). These compounds elicit behavioural responses relevant to host location, such as an increase in the duration or intensity of searching (Wajnberg and Colazza 2013). The semiochemistry of plant–herbivore interactions has also been shown to influence the ability of parasitoids to locate hosts (Conti et al. 2010). A better understanding of the chemical cues exploited by parasitoids could improve the efficacy of biological control agents by helping to select the most appropriate strain of an agent based on its performance locating hosts (Barratt et al. 2018). Results from chemical ecological studies could also be used to complement pre-release host specificity testing. For example, results from experiments which demonstrate how parasitoids sense and respond to specific volatile compounds or blends would provide valuable insights into the likelihood of an agent detecting and pursuing a non-target species, relative to its target host (Cingolani et al. 2019).

Gas chromatography coupled with electroantennographic detection (GC-EAD) can be used to identify olfactory-active compounds in a mixture, such as a solvent extract or headspace sample taken from hosts or the plants they feed on (Arn et al. 1975). In the context of biological control programmes, this technique aids in the identification of specific semiochemicals which are attractive to parasitoids (Boyle et al. 2020). The application of GC-EAD to understanding the chemical nature of attraction between biological control agents and their target hosts is well established (Olsson and Hansson 2013). However, the method is rarely applied to the chemistry mediating interactions between candidate biological control agents and non-target hosts during pre-release host specificity testing. Comparing the differences in volatile profiles between larger numbers of target or non-target taxa would help to uncover more general patterns of host preference within certain groups (Boyle et al. 2020). These kinds of insights would be useful for decision-makers when assessing the suitability of parasitoids for release, especially when these decisions would otherwise be made based solely on laboratory oviposition testing (Saunders et al. 2022), or when it is too difficult or time-consuming to collect or rear large numbers of non-target species for behavioural tests. Even when a non-target species is confirmed as a physiological host in laboratory testing, it is important to understand if the parasitoid will be motivated to search for the host in the field (Avila et al. 2016). Chemical ecology techniques such as GC-EAD offer meaningful contributions to understanding ecological host range during the pre-release risk assessment phase of classical biological control programmes.

*Trissolcus japonicus* Ashmead (Hymenoptera: Scelionidae) is an oligophagous egg parasitoid of pentatomid stink bugs native to East Asia (Talamas et al. 2013). It is considered to be the most promising biological control agent against the brown marmorated stink bug (BMSB; *Halyomorpha halys* Stål) (Hemiptera: Pentatomidae), a polyphagous horticultural pest native to the same regions (Lee et al. 2013), but recently invasive in the Americas and Europe (Hoebeke and Carter 2003; Wermelinger et al. 2008; Leskey and Nielsen 2018). BMSB is a high priority pest for biosecurity screening in New Zealand because it presents a serious risk to horticulture, trade, and tourism, which comprise a significant part of the national economy (Duthie 2015; Ballingall and Pambudi 2017). An application to release *T. japonicus* in New Zealand has been approved, with controls, by the Environmental Protection Authority (EPA) in the event the stink bug establishes (EPA 2018). Physiological host range testing in China has shown *T. japonicus* to be the most dominant parasitoid of BMSB (Zhang et al. 2017), while similar testing in the US and Europe has generally shown low emergence rates in non-target stink bugs (Hedstrom et al. 2017; Botch and Delfosse 2018; Lara et al. 2019; Haye et al. 2020). However, physiological host range testing in New Zealand showed that *T. japonicus* emerges from one endemic pentatomid species (*Hypsithocus hudsonae* Bergroth) and two native species at proportions similarly high to BMSB (*Cermatulus nasalis nasalis* Westwood and *Glaucias amyoti* Dallas), and at rates between 70–80% from two exotic non-target species (*Dictyotus caenosus* Westwood and *Monteithiella humeralis* Walker) (Charles et al. 2019; Saunders et al. 2021, 2022). While physiological host range testing has confirmed most non-target New Zealand pentatomid species as physiological hosts, uncertainty remains over the likelihood of *T. japonicus* detecting and pursuing these hosts in the field.

The host-parasite complex of pentatomid bugs and their *Trissolcus* parasitoids in New Zealand is depauperate and restricted to eight species of pentatomids and two known parasitoids (Cumber 1964; Larivière 1995; Todd et al. 2020). The New Zealand Pentatomidae consists of the introduced predatory bug *Oechalia schellenbergii* (Guérin), and the native *Cermatulus nasalis* Westwood which is split into three subspecies: the endemic subspecies *C. nasalis hudsoni* Westwood and *C. nasalis turbotti* Westwood, and the native subspecies *C. nasalis nasalis* Westwood. Herbivorous species are represented by the endemic *Hypsithocus hudsonae* Bergroth; the native species *Glaucias amyoti* Dallas; and the introduced species *Dictyotus caenosus* Westwood, *Monteithiella humeralis* Walker, *Cuspicona simplex* Walker, and *Nezara viridula*. *Trissolcus basalis* Wollaston was introduced to New Zealand in 1949 as a biological control agent against *N. viridula* (Cumber 1949, 1950). It is regarded as an effective biocontrol agent against its target host but is known to attack most non-target pentatomids (Cumber 1964). *Trissolcus oenone* Johnson is a native pentatomid parasitoid which has also been recorded attacking most species of New Zealand pentatomids (Cumber 1964; Johnson 1991). Other than an Australian captive rearing study (James and Warren 1991), *T. oenone* has not been the subject of any research to date.

A better understanding of how parasitoids perceive host semiochemicals would offer clues to their likely host-specificity (Park et al. 2018). Scelionid egg parasitoids are known to exploit a variety of chemical cues associated with different life stages of their pentatomid hosts and the plants they feed on (Austin et al. 2005; Fatouros et al. 2008; Conti and Colazza 2012). Adult stink bugs leave kairomones behind on surfaces they walk over (Colazza et al. 1999), and their feeding or oviposition activity can induce plants to release synomones which are attractive to parasitoids (Colazza et al. 2009; Salerno et al. 2019). However, few studies have identified the compounds responsible for eliciting these behavioural responses (Weber et al. 2017b). In this study, we used GC-EAD to measure antennal responses to chemical compounds associated with non-target New Zealand stink bugs in three *Trissolcus* species: *Trissolcus japonicus*, *T. basalis*, and *T. oenone*. Our primary objective was to describe the volatile compound profiles for each New Zealand stink bug species and to identify which of these compounds elicit antennal responses in each of the three parasitoid species. We hypothesised that the volatile profile for each pentatomid species would differ both qualitatively and quantitatively, and that each of the three parasitoid species would respond to different compounds based on their known host associations.

## Materials and Methods

### Insect Colonies

Pentatomid colonies were established from wild specimens and housed in clear plastic containers (~ 170 mm H × 210 mm L × 135 mm W) with ventilated lids and maintained in a temperature controlled room between 20–25°C (16:8 h L:D), depending on demand for egg masses. Pentatomids were provisioned with moist cotton, wax paper for oviposition, and food after nymphs had moulted to second instar. We provided *Pittosporum spp.* and *Coprosma spp.* fruits for *M. humeralis* and *G. amyoti*, *Solanum spp.* fruits and tomatoes for *C. simplex*, green beans and raw peanuts for *N. viridula*, *Plantago spp.* seed heads for *D. caenosus*, and *Spodoptera litura* (F.) larvae from an existing laboratory culture for *C. nasalis nasalis*, *C. nasalis hudsoni*, and *O. schellenbergii*. We reared *H. hudsonae* on different combinations of food but were ultimately unsuccessful in rearing eggs through to ovipositing adults (Saunders et al. 2021). *Cermatulus nasalis turbotti* was excluded due to the difficulty of collecting specimens.

*Trissolcus basalis* was originally collected in naturally laid eggs of wild *N. viridula* on *Cleome spinosa* Jacq. from Kelmarna Gardens, Auckland, in February 2019. This colony was reared through approximately 15 generations on *N. viridula* in the laboratory before being used in experiments. *Trissolcus oenone* was originally collected in naturally laid eggs of *C. simplex* on *Coprosma spp.* (near *Solanum spp.*) from the suburb of Mt Albert, Auckland, in November 2019. This colony was reared through approximately three generations in the lab on *C. simplex* before being used for experiments. Both parasitoid colonies were reared on their original hosts in a temperature controlled room between 18–25°C (16:8 h L:D), depending on the need to time emergence with EAD recordings. Fresh pentatomid eggs (< 24h old), or eggs stored at 10°C for no longer than two weeks, were used to maintain the colonies.

Shipments of *T. japonicus* were sourced from the USDA-ARS Beneficial Insect Research Unit in Newark, Delaware, and imported into Plant & Food Research containment facilities in Auckland or Lincoln, Canterbury, for use in experiments. Shipments consisted of parasitised BMSB egg masses held in individual 10-dram plastic vials. Egg masses were kept between 18–25°C while parasitoids emerged and mated, depending on the need to time emergence with pentatomid egg production.

### Extract Preparation and Chemical Analysis

Solvent stink bug extracts were used to identify compound profiles for each stink bug species, and to measure parasitoid antennal responses to compounds within each extract. For each of the five extract replicates, four female stink bugs were taken from colony containers and immersed in 1 ml of hexane for five minutes inside a glass vial. Each millilitre of hexane contained 10 µg each of n-decane and ethyl tetradecanoate as internal standards. All stink bugs were assumed to have been mated and were taken from cages where eggs were being laid. The extract was then transferred to a clean glass vial and kept at -20°C until required for analysis. Each extract was analysed on a gas-chromatograph (GC, Agilent 7890B) coupled to a mass-spectrometer (MSD, Agilent 5977A). A 1 µl sample was injected into the GC in splitless mode and carried by helium gas at a flow rate of 1.6 mL/min. The GC column was non-polar (Agilent DB-5 ms) and measured 30m × 0.25mm ID with 0.25μm film thickness. The temperature program started at 40°C and was held for 2 min, then increased to 250°C at a rate of 4°C/min, followed by a 10°C/min ramp to 280°C, where it was held for 10 min. The transfer line was kept at 250°C.

### Electrophysiological Recordings

We used GC-EAD to identify olfactory-active volatile compounds from stink bug extracts on the antennae of all three *Trissolcus* species. For each pentatomid species tested, we anaesthetised five females from each parasitoid species with carbon dioxide gas before removing their heads and the distal tips of one of their antennae with a fine scalpel under a stereomicroscope. Each specimen was positioned between two silver wire electrodes sheathed by glass capillaries pulled to fine points. Glass capillaries were trimmed with a ceramic cutter and filled with Ringer’s solution (Kaissling 1995). The excised end of the head was positioned into contact with the reference electrode and the excised antenna was positioned into contact with the recording electrode, using a motorised micromanipulator (MP-225, Sutter Instrument Co., USA). The GC transfer-line was connected to a glass airflow tube containing a charcoal-filtered and humidified air stream with a flow rate of 400 ml/min. The specimen preparation was positioned in front of the humidified air stream containing the column effluent. We used an Agilent 7890A GC with a 30 m × 0.32 mm ID HP-5 capillary column with a film thickness of 0.25 µm (Agilent Technologies, CA, USA). The GC was equipped with a flame ionisation detector (FID) and the column flow was split in a 1:1 ratio between the FID and antennal preparation. The output was amplified using an intelligent data acquisition controller (IDAC 4, Ockenfels Syntech, Germany) and manipulated using Autospike software (v3.9, Syntech, Germany). We injected 1 µl samples of extract into the GC injection port set to 250°C in splitless mode. Samples passed through the column at 1 mL/min and were carried by helium. The FID was set to 300°C, while the GC oven temperature was programmed to start at 60°C and held for 1 min, before increasing to 280°C at a rate of 20°C/min, where it was held for 10 min. We used Autospike software (v3.9, Syntech, Germany) to record the FID response as compounds eluted from the GC, and simultaneously, to record the insect’s antennal response to each compound. We used a different parasitoid for each recording and aimed to capture at least five clear recordings showing consistent responses for each stink bug extract with each parasitoid species. For each response, we measured the amplitude of each depolarisation event manually inside Autospike.

Once we identified responsive compounds in the stink bug extracts, we conducted another round of GC-EAD recordings with synthetic standards to confirm responsive compounds. We injected 1 µl samples of a solution containing synthetic standards of identified compounds (each at a concentration of 0.1 mg/ml) for each parasitoid species. Next we presented individual test compound to each parasitoid using electroantennogram (EAG) recordings to identify antennal responses in at least three recordings. We applied a 10-μL aliquot of each compound solution (at a concentration of 100 mg/ml) to a 5 × 25 mm strip of filter paper (Whatman No. 1; Whatman, UK) and allowed the solvent to evaporate for 10 s before placing the paper inside a glass Pasteur pipette (146 mm; Fisher Scientific Co., Pittsburgh, Pennsylvania) to form an odour cartridge. The pipette tip was inserted into a 2-mm diameter hole in the glass airflow tube 10 cm from the outlet. A 0.1-s pulse of charcoal-filtered air (10 mL s^−1^) was passed through the wide end of the pipette to carry a puff of compound into the airflow tube and over the insect antenna, using an electronic airflow controller (CS-55; Syntech, Germany). We presented each test compound three times in succession with at least 30 s of time between successive stimulations. At the start of each EAG recording with *T. basalis* and *T. oenone* we presented a blank air cartridge and a series of solvent puffs. Before and after presenting test compounds, and after every six compound puffs, we presented a single puff of (*E*)-2-decenal to act as a standard response to allow for the normalisation of responses. We wrapped the wide end of pipettes in aluminium foil when not in use to minimise evaporation of test compound, and we used each cartridge for less than 10 puffs. For the control air cartridge, we kept the filter paper blank, and for the solvent control cartridges, we applied a 10-μL aliquot of neat hexane or acetone.

Synthetic standards were obtained as follows: (*E)*-2-decenal, (*E*)-2-octenal, (*E*)-2-decenyl acetate, *n*-decane, (*E*)-2-hexenal, *n*-tridecane, *n*-nonadecane and ethyl tetradecanoate were purchased from Sigma Aldrich (St. Louis, MO, USA); and (*E*)-4-oxo-2-hexenal was synthesised at The New Zealand Institute for Plant & Food Research, Palmerston North, by Barry Bunn.

### Data Analysis

To identify stink bug compounds, we analysed solvent extracts in a GC–MS using MassHunter WorkStation 2015 and the NIST Mass Spectral Search Program 2.4 2020. The NIST library matches were confirmed by calculating the Kovats retention index (KI) (Kováts and Weisz 1965) of each compound by running a hydrocarbon series (C8 to C28) using the same temperature program and column type as the extract experiments. We further confirmed compounds by analysing a solution of each of the synthetic compounds against the extracts by comparing the retention time and mass spectral patterns. To compare the volatile profiles of each pentatomid species, we quantified the compounds using the internal standard method and calculated the proportion of each active compound based on peak area in each stink bug extract versus peak area of the internal standards.

Further analyses were conducted in R 4.0.2 (R Core Team 2020). To compare the similarity of each pentatomid species based on their volatile compound profiles, we performed nMDS with the quantified compound values and plotted the results in an ordination plot. We excluded *O. schellenbergii* as these extracts had few olfactory-active compounds present. To compare the magnitude of electrophysiological responses between the three parasitoid species, we calculated mean responses across replicates recorded in the two rounds of GC-EAD experiments. For EAG recordings, we normalised responses in relation to five standard responses to (*E*)-2-decenal (a compound known to be responsive) obtained throughout each recording.

## Results

**Fig. 1:**
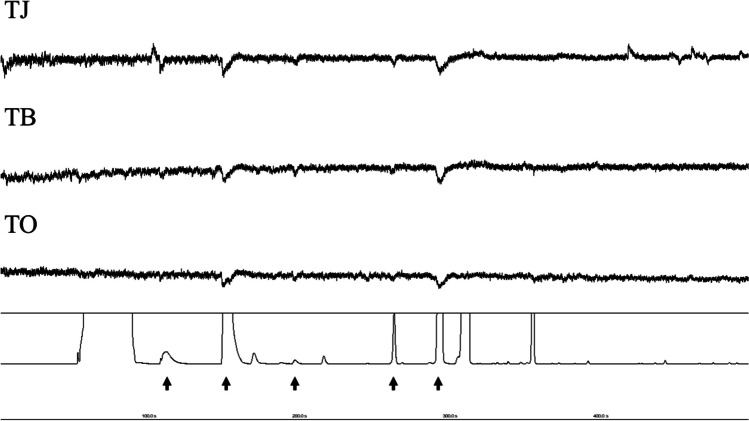
Representative GC-EAD recording showing responses from *T. japonicus* (TJ), *T. basalis* (TB), and *T. oenone* (TO) to stink bug solvent extract (in this example *Cuspicona simplex*). Arrows show responsive compound peaks, from left: (*E*)-2-hexenal, (*E*)-4-oxo-2-hexenal, (*E*)-2-octenal, *n*-dodecane, (*E*)-2-decenal. The full set of recordings, along with all Autospike files, is available for download (see data availability statement)

**Fig. 2:**
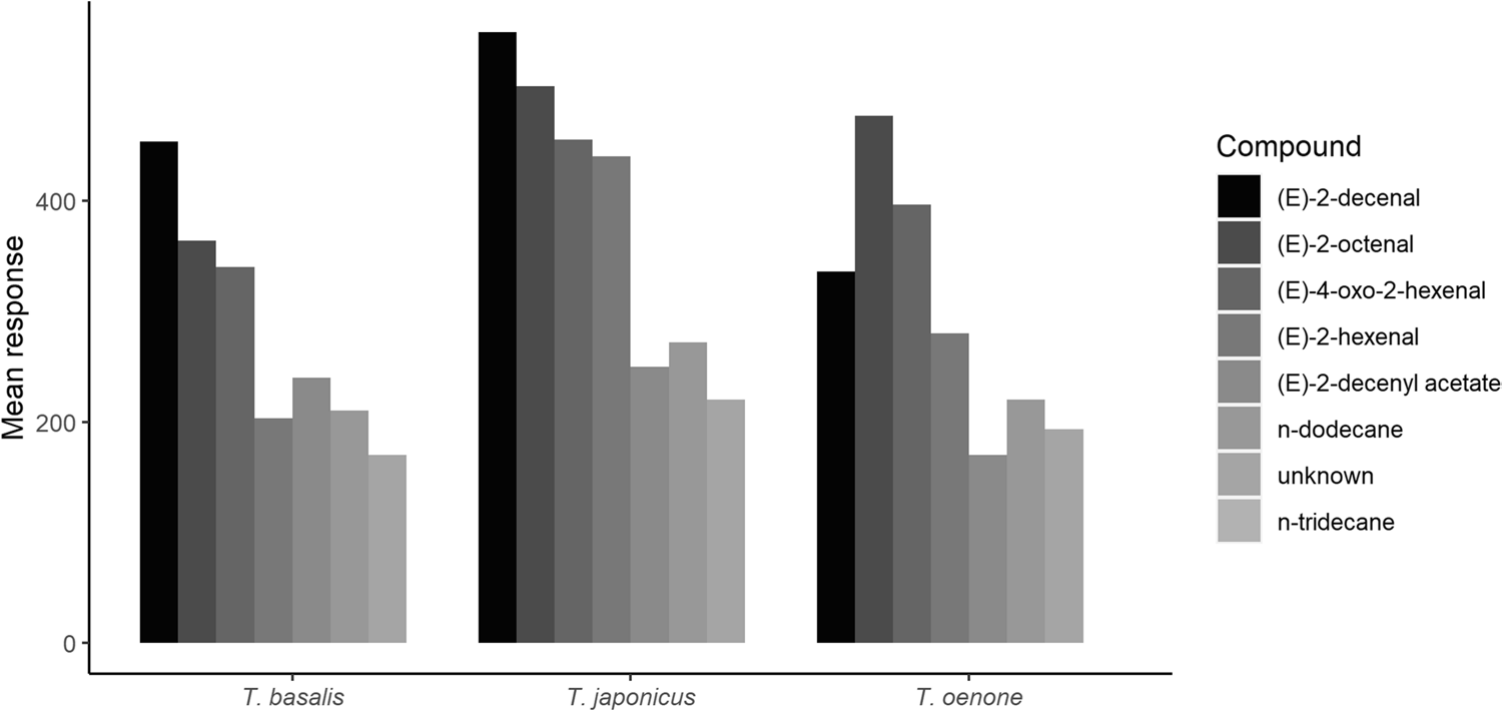
Mean absolute GC-EAD responses to bioactive compounds in stink bug solvent extracts

**Fig. 3:**
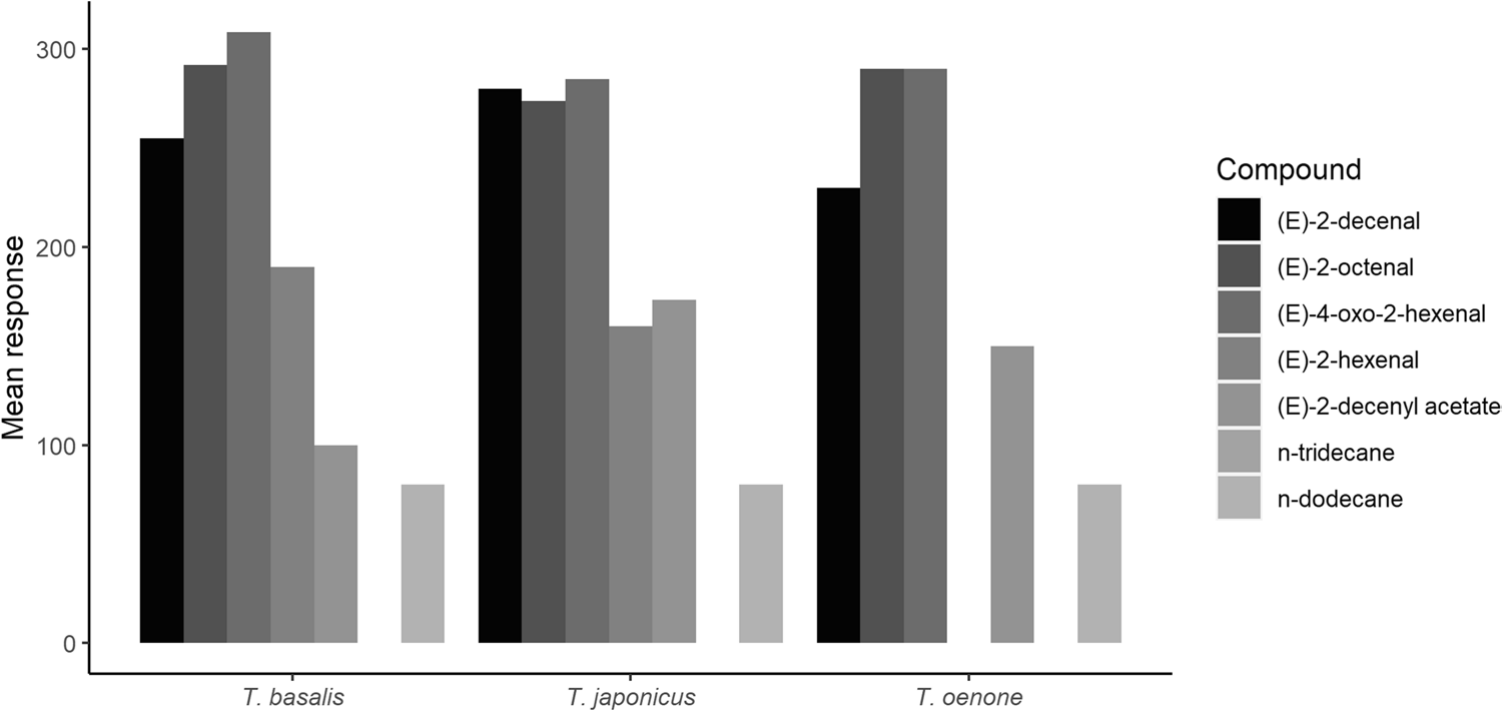
Mean absolute GC-EAD responses to synthetic standards of bioactive compounds identified from solvent extracts

From the combination of GC-EAD and EAG experiments, we identified a total of seven compounds which elicited antennal responses from parasitoids: (*E*)-2-hexenal, (*E*)-2-octenal, (*E*)-4-oxo-2-hexenal, (*E*)-2-decenal, *n*-dodecane, *n*-tridecane, and (*E*)-2-decenyl acetate (Table 1). In GC-EAD experiments with stink bug solvent extracts, seven compounds elicited clear antennal responses from at least one parasitoid species (Figs. 1 and 2). This included responses to an unknown compound in extracts of *H. hudsonae*, *D. caenosus*, and *N. viridula* with a KI of 1204, eluting between *n*-dodecane and (*E*)-2-decenal, but we were unable to identify it based on mass spectra. All three parasitoids responded most strongly to (*E*)-4-oxo-2-hexenal, but responses to (*E*)-2-decenal and (*E*)-2-octenal were also very strong. In the second round of GC-EAD experiments, where each parasitoid was exposed to individual synthetic compounds identified during the previous step, we confirmed responses to all six successfully identified compounds from the first round of EAD (Fig. 3).

**Table 1:** Quantified compound (ng) per 1µl of stink bug extract for nine New Zealand Pentatomidae. Values for unknown compound (*) represent mean percent area of GC peaks in lieu of authentic compound

Compound	Kovats Index	*Cermatulus nasalis hudsoni*	*Cermatulus nasalis nasalis*	*Cuspicona simplex*	*Dictyotus caenosus*	*Glaucias amyoti*	*Hypsithocus hudsonae*	*Monteithiella humeralis*	*Nezara viridula*	*Oechalia scellenbergii*
(*E*)-2-hexenal	856	9.4	13.79	593.89	93.37	583.54	321.22	260.34	442.28	0
(*E*)-4-Oxo-2-hexenal	960–971	93.74	281.84	294.24	127.18	332.04	151.82	28.16	171.91	0
(*E*)-2-octenal	1056–1060	0	5.21	7.75	115.91	12.65	106.88	9	108	0
*n*-dodecane	1195–1200	2.02	1.61	19.32	7.43	42.19	4.01	0	14.83	0
Unknown*	1204–1209	0.41	0.49	0	17	0.27	19.18	0.16	0.41	0
(*E*)-2-decenal	1260–1271	8.96	46.05	354	0	409.62	12.01	374.39	215.58	0
*n*-tridecane	1286–1311	59.01	65.73	427.06	164.04	703.97	124.6	492.76	301.81	2.11
(*E*)-2-decenyl acetate	1401–1407	98.8	224.84	93.59	0	312.61	34.87	142.59	145.72	0

All three parasitoids responded most strongly to (*E*)-2-decenal, (*E*)-2-octenal, (*E*)-4-oxo-2-hexenal, while responses to (*E*)-2-decenyl acetate and *n*-dodecane were generally weaker. *Trissolcus oenone* failed to respond to synthetic (*E*)-2-hexenal.

**Fig. 4:**
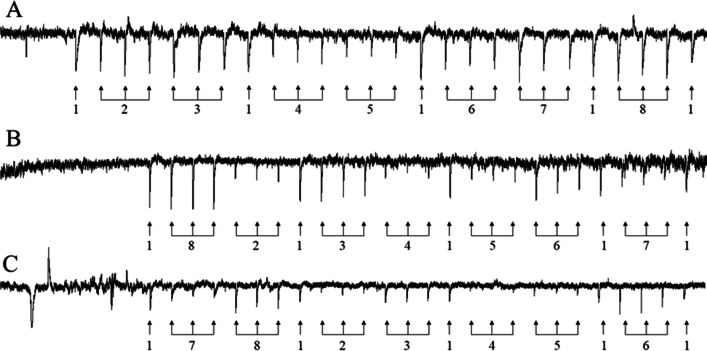
Representative EAG recordings with each parasitoid and each compound identified as bioactive from GC-EAD experiments. **A** *Trissolcus japonicus*. **B** *Trissolcus basalis*. **C** *Trissolcus oenone*. 1. (*E*)-2-decenal (standard). 2. n-dodecane. 3. (*E*)-2-decenal. 4. *n*-tridecane. 5. (*E*)-2-decenyl acetate. 6. (*E*)-2-hexenal. 7. (*E*)-4-oxo-2-hexenal. 8. (*E*)-2-octenal

**Fig. 5:**
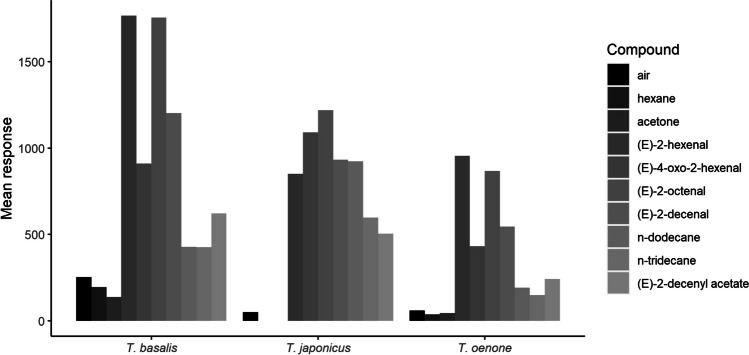
Normalised mean EAG responses to bioactive compounds presented to parasitoids

In EAG experiments, relative parasitoid responses were broadly similar to those obtained in GC-EAD recordings, although responses to (*E*)-4-oxo-2-hexenal for *T. basalis* and *T. oenone* were slightly weaker than expected, based on GC-EAD results (Figs. 4 and 5). In *T. japonicus*, there was a less pronounced difference between the group of four compounds which elicited higher responses ((*E*)-2-decenal, (*E*)-2-octenal, (*E*)-4-oxo-2-hexenal, and (*E*)-2-hexenal) and the group of three compounds which elicited lower responses ((*E*)-2-decenyl acetate, *n*-tridecane, and *n*-dodecane) compared to the other two parasitoids.

**Fig. 6:**
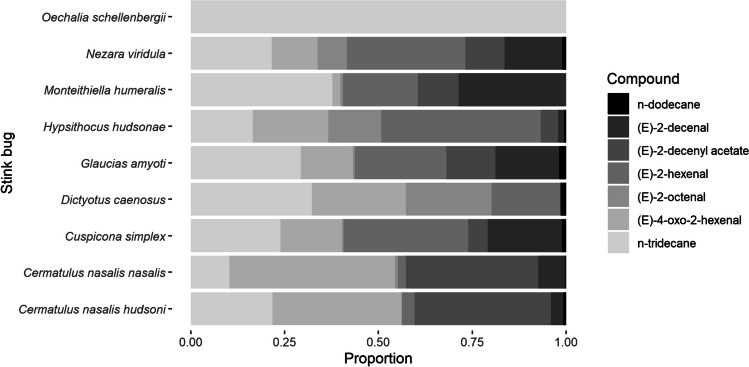
Proportion of bioactive volatile compounds making up stink bug solvent extracts

**Fig. 7:**
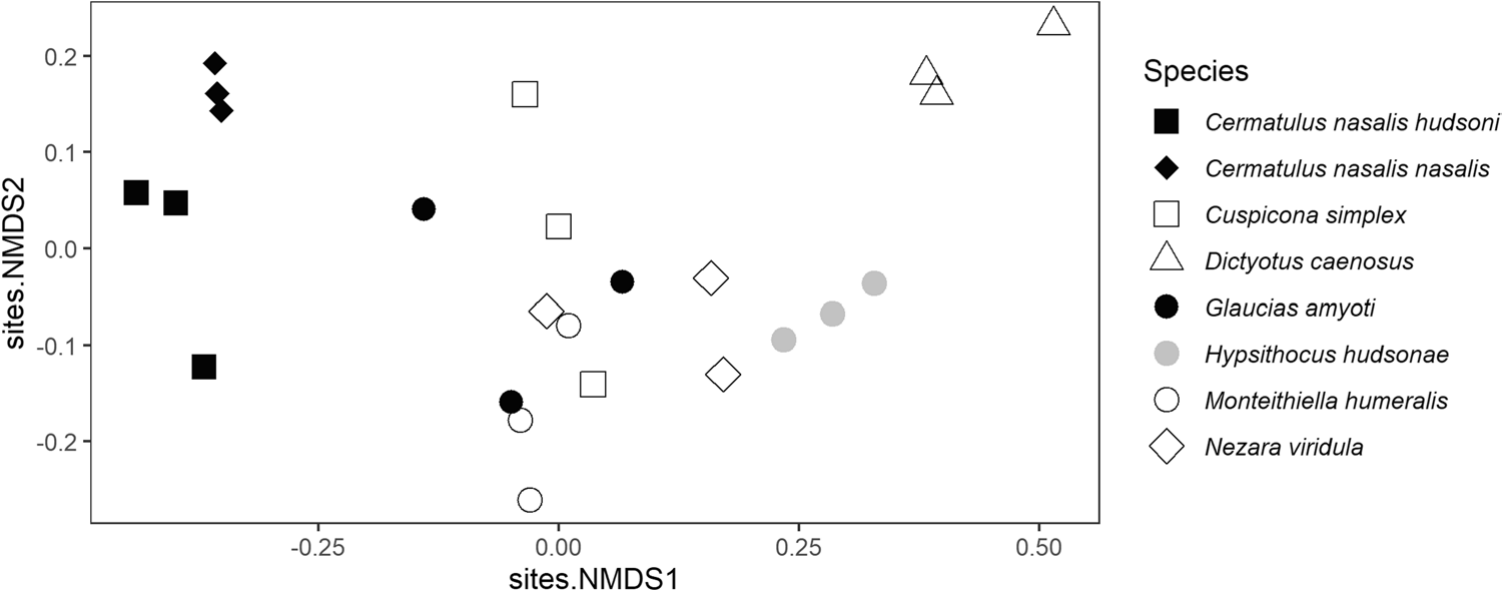
Non-metric Multidimensional Scaling (nMDS) plot showing similarity of pentatomid extract samples based on their volatile profiles, with native or endemic taxa represented by filled shapes

The comparison of bioactive compounds making up the volatile profile of each pentatomid species showed that most of the seven bioactive compounds were detected within most of the pentatomid extracts, although the relative amounts differed considerably (Fig. 6). The only major compound detected in *O. schellenbergii*, which was also found in all pentatomid species, was *n*-tridecane. Based on the ordination of these results, the two predatory subspecies of *C. nasalis* appeared to form a cluster, while the introduced *D. caenosus* had the least similar profile to other species. The remaining species (*C. simplex*, *M. humeralis*, *N. viridula*, and *G. amyoti*) all overlapped to some degree in the similarity of their volatile profiles, while the extracts of the endemic *H. hudsonae* formed a satellite cluster to this group, appearing to be most similar to *N. viridula* (Fig. 7).

## Discussion

We conducted multiple rounds of GC-EAD and EAG experiments to identify seven compounds associated with New Zealand pentatomids which elicited antennal responses in three different species of *Trissolcus* parasitoids: (*E*)-2-decenal, (*E*)-2-octenal, (*E*)-4-oxo-2-hexenal, (*E*)-2-hexenal, (*E*)-2-decenyl acetate, n-dodecane, and n-tridecane. Previous work on the chemical ecology of the Pentatomidae has shown that adult stink bugs produce a range of compounds in their metathoracic glands, and that these compounds can act as defensive allomones and/or pheromones, depending on the receiver (Aldrich 1988). Common components of defensive secretions include short chain alcohols, aldehydes, and alkanes, and the blend of compounds can change significantly depending on life stage (Borges and Aldrich 1992; Eliyahu et al. 2012). Adults of most stink bug species appear to share much of their defensive chemistry across different genera, with the more common compounds including short chain alcohols and their esters (e.g. (*E*)-2-decenyl acetate), aldehydes (e.g. (*E*)-2-alkenals and 4-oxo-(*E*)-2-alkenals), and linear hydrocarbons, of which* n*-tridecane is reported as one of the most commonly found, and most abundant (Aldrich 1995; Millar 2005; Weber et al. 2017a). Brown marmorated stink bug is known to produce all seven of the compounds identified from extracts of New Zealand Pentatomidae, and in fact, the seven compounds we identified constitute the major components in BMSB volatile profiles (Zhong et al. 2017; Nixon et al. 2018).

The stink bugs we tested largely overlapped in the qualitative composition of their volatile profiles, although the quantities of compounds differed for each species. The two predatory subspecies had the most similar volatile profiles, while perhaps unusually, the endemic alpine species, *H. hudsonae*, appeared to be most similar to the cosmopolitan pest *N. viridula*. We originally hypothesised that each pentatomid species would have different compounds and different ratios of compounds in its volatile profile, and that each of the three parasitoid species may respond to different compounds based on their physiological host range. While we expected more variation in the blends of volatile compounds making up New Zealand stink bug extracts, the variation present appeared to at least suggest our sampling technique was capable of detecting both qualitative and quantitative differences in volatile blends between species. For example, the two predatory subspecies of *C. nasalis* would be expected to cluster together in their volatile profile based on their close taxonomic relationship, and they did indeed display very similar chemical profiles for the active compounds we identified. It remains unclear why the *O. schellenbergii* extract had far fewer compounds than the other pentatomid extracts, as we used the same methodology to extract compounds across all species. Aldrich et al. (1996) were able to identify compounds from male *O. schellenbergii* dorsal abdominal glands, including (*E*)-2-hexenal, by dissecting the glands and standing in dichloromethane overnight, but no other work on the chemistry of this species has been reported.

Scelionid egg parasitoids are known to be able to discriminate between different hosts based solely on the compounds that adult stink bugs leave behind on a substrate (Conti et al. 2004; Colazza et al. 2009; Frati et al. 2013). Our results suggest the differing ratios of compounds associated with stink bugs may be enough for these parasitoids to separate stink bug species, and therefore express host preferences. *Trissolcus basalis* is known to be attracted to (*E*)-2-decenal and (*E*)-2-hexenal, which are produced in relatively large quantities by the nymphs and adults of many stink bugs (Mattiacci et al. 1993; Laumann et al. 2009). *Telenomus podisi* (F.) is attracted to (*E*)-2-hexenal (Vieira et al. 2014), but because this compound is a relatively common plant volatile produced in large amounts by certain crops, it remains unclear whether this parasitoid is using the compound as a cue associated with plants, hosts, or both (Moraes et al. 2008). Both *T. basalis* and *T. podisi* are known to be attracted to (*E*)-4-oxo-2-hexenal (Laumann et al. 2009), and while this compound is relatively common in the Hemiptera, 4-oxo-(*E*)-2-alkenals have never been found to be associated with any other insects (Millar 2005). *Trissolcus basalis* is known to be attracted to (*E*)-4-oxo-2-hexenal (Laumann et al. 2009). Our results suggest the ability for *Trissolcus* parasitoids to detect compounds associated with stink bugs is highly conserved, as all three species showed antennal responses to the same compounds despite widely differing native ranges, and few shared natural hosts between them. It would be worth exploring if quantitative similarities in volatile profiles translates to differences in searching motivation in open arena arrestment bioassays (Conti et al. 2004).

Zhong et al. (2017) recently conducted GC-EAD recordings with female *T. japonicus* in relation to solvent extracts made from BMSB females. They showed that female BMSB volatile profiles contain the same seven compounds we found to be bioactive in the three parasitoids we tested, and in BMSB these seven compounds are all major peaks. However, they reported *T. japonicus* antennal responses to just two compounds: *n*-tridecane and (*E*)-2-decenal. They also reported parasitoid attraction to *n*-tridecane and parasitoid aversion to (*E*)-2-decenal in Y-tube olfactometer experiments. We only observed antennal responses to *n*-tridecane when puffing single compounds over the antennae (likely at higher concentrations than occur naturally), and even then, it was always one of the weakest responses for all three *Trissolcus* species we tested. More recently, Malek et al. (2021) investigated *T. japonicus* arrestment responses in open arenas contaminated with BMSB and the suboptimal host *Podisus maculiventris* (Say). While they observed motivated searching behaviour from parasitoids in response to footprint compounds from both species, parasitoids spent longer searching for BMSB, and stink bug trails continued to elicit responses in parasitoids 72 h after they were deposited. GC–MS analyses revealed *n*-tridecane and (*E*)-2-decenal were deposited by stink bugs, and a 4:1 blend of these compounds prolonged residence times of parasitoids in open arenas while (*E*)-2-decenal alone reduced searching activity. The combination of *n*-tridecane and (*E*)-2-decenal likely have a kairomonal effect on *T. japonicus*, although there are potentially other compounds which influence the host-preferences or host-finding ability of this parasitoid. There is evidence to suggest that linear hydrocarbons instead act as synergists to promote either the evaporation of defensive blends, or the penetration of these blends into the cuticles of other insects, rather than acting as kairomones alone (Eliyahu et al. 2012; Weber et al. 2017b). However, the lack of chemical ecological studies on scelionid egg parasitoids makes it difficult to draw any firm conclusions at this stage. While the study of kairomone-mediated host searching in egg parasitoids of pentatomids has started to reveal the identities of attractive compounds associated with stink bugs (Fatouros et al. 2008; Conti and Colazza 2012; Weber et al. 2017a), our understanding of which compounds are attractive or repulsive to scelionid egg parasitoids is still developing, due in large part to a paucity of electrophysiological studies to confirm the identities of behaviourally relevant compounds.

We ran multiple GC-EAD and EAG experiments with stink bug solvent extracts and synthetic standards to identify compounds that may have a kairomonal effect on *Trissolcus* egg parasitoids. It was only during EAG experiments that we observed responses to *n*-tridecane. These compounds can now be screened more thoroughly in behavioural bioassays to understand how they influence the searching behaviour of parasitoids. Our results show the utility of applying chemical ecological techniques to understand the chemical basis of attraction between parasitoids and hosts. A more accurate understanding of the kairomonal activity of specific compounds associated with target and non-target hosts will help to forecast risks associated with the introduction of classical biological agents. These kinds of techniques complement traditional oviposition tests, and are able to provide useful information about the ability of parasitoids to detect certain hosts, and their motivation to search for hosts based on chemical cues (Conti et al. 2004; Cingolani et al. 2019). Ultimately, a better understanding of how semiochemistry mediates host preferences in parasitoids should lead to improved pre-release host range testing procedures and better predictions of non-target risks before agents are released.

## Data Availability

All data generated during this study is available on Zenodo (10.5281/zenodo.6634031).
